# Termination of Vernakalant-Resistant Atrial Fibrillation by Inhibition of Small-Conductance Ca^2+^-Activated K^+^ Channels in Pigs

**DOI:** 10.1161/CIRCEP.117.005125

**Published:** 2017-10-17

**Authors:** Jonas Goldin Diness, Lasse Skibsbye, Rafel Simó-Vicens, Joana Larupa Santos, Pia Lundegaard, Carlotta Citerni, Daniel Rafael Peter Sauter, Sofia Hammami Bomholtz, Jesper Hastrup Svendsen, Søren-Peter Olesen, Ulrik S. Sørensen, Thomas Jespersen, Morten Grunnet, Bo Hjorth Bentzen

**Affiliations:** From the Acesion Pharma, Copenhagen, Denmark (J.G.D., R.S.-V., C.C., D.R.P.S., S.H.B., U.S.S., M.G., B.H.B.); Department of Biomedical Sciences, Faculty of Health and Medical Sciences, University of Copenhagen, Denmark (L.S., J.L.S., P.L., D.R.P.S., S.-P.O., T.J., M.G., B.H.B.); and the Heart Centre, Rigshospitalet, Copenhagen, Denmark (J.H.S.).

**Keywords:** atrial fibrillation, blood pressure, heart atria, swine, vernakalant

## Abstract

**Background:**

Evidence has emerged that small-conductance Ca^2+^-activated K^+^ (SK) channels constitute a new target for treatment of atrial fibrillation (AF). SK channels are predominantly expressed in the atria as compared with the ventricles. Various marketed antiarrhythmic drugs are limited by ventricular adverse effects and efficacy loss as AF progresses.

**Methods and Results:**

A total of 43 pigs were used for the studies. AF reversion in conscious long-term tachypaced pigs: Pigs were subjected to atrial tachypacing (7 Hz) until they developed sustained AF that could not be reverted by vernakalant 4 mg/kg (18.8±3.3 days of atrial tachypacing). When the SK channel inhibitor AP14145 was tested in these animals, vernakalant-resistant AF was reverted to sinus rhythm, and reinduction of AF by burst pacing (50 Hz) was prevented in 8 of 8 pigs. Effects on refractory period and AF duration in open chest pigs: The effects of AP14145 and vernakalant on the effective refractory periods and acute burst pacing-induced AF were examined in anaesthetized open chest pigs. Both vernakalant and AP14145 significantly prolonged atrial refractoriness and reduced AF duration without affecting the ventricular refractoriness or blood pressure in pigs subjected to 7 days atrial tachypacing, as well as in sham-operated control pigs.

**Conclusions:**

SK currents play a role in porcine atrial repolarization, and pharmacological inhibition of these with AP14145 demonstrates antiarrhythmic effects in a vernakalant-resistant porcine model of AF. These results suggest SK channel blockers as potentially interesting anti-AF drugs.

## Abstract 2

Supplemental Digital Content is available in the text.

**See Editorial by Heijman and Dobrev**

**Figure:**
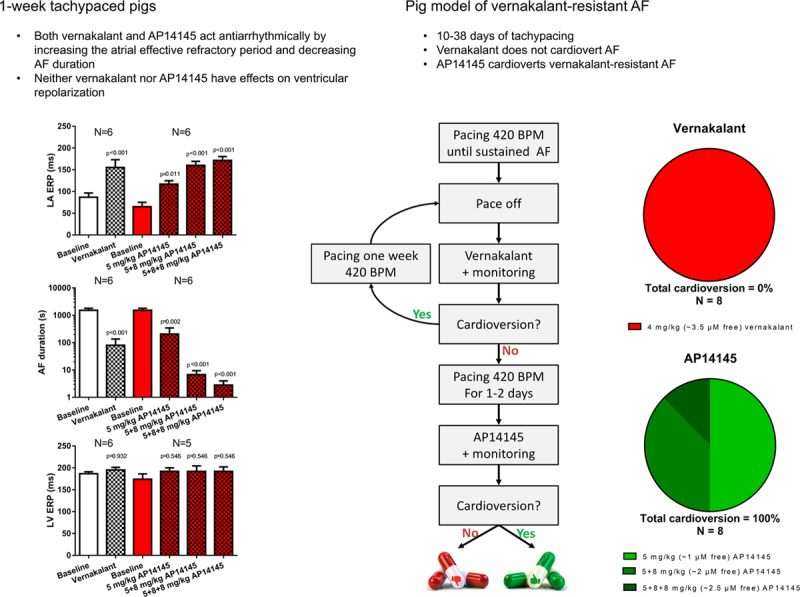

**WHAT IS KNOWN?**

- Small-conductance Ca^2+^-activated K^+^ (SK) channels are predominantly expressed in the atria as compared with the ventricles.
- Various marketed antiarrhythmic drugs are limited by ventricular adverse effects and efficacy loss as atrial fibrillation progresses.

**WHAT THE STUDY ADDS?**

- Data from a newly developed porcine model of vernakalant-resistant atrial fibrillation showing that SK channel currents play a role in porcine atrial repolarization.
- Data showing that pharmacological inhibition of SK channel current with AP14145 demonstrates antiarrhythmic effects in a vernakalant-resistant porcine model of atrial fibrillation.

Effective, safe, and tolerable pharmacological treatment for atrial fibrillation (AF) remains an unmet need. The latest medication to reach the market for intravenous cardioversion was the combined sodium and potassium channel inhibitor vernakalant, however not yet available in the United States. Vernakalant terminated ≈50% of episodes of AF lasting <7 days in randomized controlled studies, with its highest conversion rate during the first few days while after 8 to 45 days of AF, the conversion rate was <10%, which was not statistically different from that of placebo.^1,2^

If lasting for >24 hours, AF promotes further progression of the disease—a phenomenon described as AF begets AF.^3^ If atrial remodeling continues, AF often progresses to more sustained forms and becomes more resistant to both pharmacological and nonpharmacological treatments, including ablation.^4–6^ Among contributing factors, an increased influx of calcium seems to promote fibrosis development and remodeling.^7^

Three subtypes of small-conductance calcium-activated K^+^ channels, SK1, SK2, and SK3, are represented in the heart. They are all exclusively activated by intracellular Ca^2+^, and the current conducted by these channels is recognized as *I*_KCa_. Originally, SK channels were cloned from the central nervous system where they contribute to the afterhyperpolarization of the neuronal action potential.^8^ With the finding of SK channels in cardiac tissue, it was demonstrated that these channels are also important for the repolarization of cardiac action potentials.^8–11^ Confirmation of the role of SK channels in human AF has since been provided in several genome-wide association studies concluding that common variants in the genes encoding SK2 and SK3 are associated with AF.^12–14^ We demonstrated that *I*_KCa_ inhibition can terminate AF or protect against its induction in models of AF in isolated perfused heart preparations from rat, guinea pig, and rabbit, as well as in in vivo models of AF in rat, dog, and horse.^8–11,15^ We also obtained evidence that SK channels play a functional role in right atrial (RA) appendage tissue, but not ventricular tissue, from patients in sinus rhythm (SR), as well as patients with >6 months of AF, and that inhibition of *I*_KCa_ with the SK channel inhibitor NS8593 prolonged refractory periods in atrial tissue from these patients.^16^ Preliminary evidence suggests that the *I*_KCa_ is increased in patients with paroxysmal AF compared with patients in SR, and *I*_KCa_ inhibition by the peptide apamin was associated with a 4-fold increased action potential prolongation in cells from patients in paroxysmal AF.^17^

Significant antiarrhythmic effects in normal and remodeled atria, without adverse effects in the ventricles, have long been a desirable, but nonachievable, therapeutic goal. This seems possible by inhibition of the SK channels, which may, therefore, qualify as a promising target for the development of a novel agent for treatment of AF.

In a preclinical canine model of vagotonic AF, vernakalant cardioverted 7 of 7 dogs at a dose of 4 mg/kg.^18^

To obtain a preclinical model better simulating clinical AF, we performed long-lasting atrial tachypacing (AT) in pigs to develop sustained AF until it was no longer possible to convert with a clinically relevant dose of vernakalant. In this model of sustained vernakalant-resistant AF, we tested whether an SK channel inhibitor, the tool compound AP14145, could successfully convert AF to SR and protect against reinduction of AF.

## Methods

### In Vitro Electrophysiology

Effects of AP14145 on hSK3 (KCa2.3) and hERG (hKV11.1) were investigated by automated patch clamp (Qpatch; Biolin Scientific, Sophion, Denmark) as previously described.^19^ Likewise, the effect on rNa_V_1.5 was evaluated as earlier described^20^ but with a modified voltage protocol so that rNa_V_1.5 currents were elicited with 1-second interval by a 50-ms depolarizing step to −20 mV from a holding potential of −120 mV (a total of 80 pulses). Effect of AP14145 on L-type calcium channels was investigated by automated patch clamping (Qpatch; Biolin Scientific) using a hCa_V_ 1.2 stable cell line (α1C, β2C, and α2delta; SB Drug Discovery, Glasgow, United Kingdom).

Off-target effects on Kir3.1/Kir3.4 (*I*_KACh_), K_V_1.5 (*I*_Kur_), K_V_7.1/KCNE1 (*I*_Ks_), K_V_4.3/KChiP2 (*I*_to_), and Kir2.1 (*I*_K1_) were investigated using 2-electrode voltage-clamp experiments on *Xenopus laevis* oocytes expressing the relevant channels.^19^ For further details, please see Data Supplement.

### Animal Models

All animal studies were performed under a license from the Danish Ministry of Environment and Food (license No. 2012-15-2934-00083) and in accordance with the Danish guidelines for animal experiments according to the European Commission Directive 86/609/EEC. A total of 43 Danish landrace pigs (from Krigsagergård w. Henrik Larsen, Gilleleje, Denmark; 12–13 weeks old, 30–35 kg gilts) were studied. The pigs were divided into 4 main groups: (1) open chest surgery (n=21), (2) cardioversion in conscious pigs (n=8), (3) expression studies (n=12), and (4) pharmacokinetic studies (n=2). The open chest surgery group consisted of pigs subjected to 1-week AT and sham-operated time matched controls (CTRL). The group for conscious cardioversion studies consisted of long-term AT pigs with persistent AF that could not be converted by a clinically relevant dose of vernakalant. The group for expression studies was divided into long-term AT pigs and control pigs.

All pigs underwent the following procedure: After pre-medication with zoletil pig mixture (250 mg dry tiletamin+zolazepam, 6.5 mL xylazine 20 mg/mL, 1.25 ketamine 100 mg/mL, 2.5 mL butorphanol 10 mg/mL, and 2 mL methadone 10 mg/mL) 0.1 mL/kg given intramuscularly, the pig was given an intravenous infusion of propofol and fentanyl (15 mg/kg per hour and 50 µg/kg per hour, respectively) and intubated and ventilated with a tidal volume of 10 mL/kg and a respiration frequency of 12 to 14 per minutes. During surgery arterial partial pressure of carbon dioxide (Paco_2_), blood pressure and ECG were monitored, and the pig was given 6 mL/kg per hour isotonic saline solution. Under aseptic conditions and fluoroscopic guidance, a bipolar pacing-electrode was inserted into the RA appendage and connected to a neurostimulator (Medtronic Synergy versitrel or Itrel 3) implanted subcutaneously in the neck region. The pigs in the long-term AT group received a central venous catheter in the internal jugular vein with an exit in the dorsal neck region.

### Open Chest Surgery

In the 1-week AT pigs, after 7 days recovery after pacemaker implantation, the pacemakers were turned on and the RA was paced 420 beats per minute (BPM) for 7 days. To avoid symptoms of heart failure caused by a high ventricular rate because of pacing, the pigs were medicated with digoxin (250 µg/d) starting 4 days before turning on the pacemaker and discontinued 2 days before open chest surgery. The same procedure was used in the CTRL pigs except that the pacemakers remained turned off in this group. Sixteen hours before study days, the pacemaker was deactivated. On study days, pigs were anesthetized as described for the pacemaker implantation. After median sternotomy, custom-made hook-shaped teflon-coated stainless steel electrodes for recording and stimulation were inserted into the left atrial (LA) appendage and the left ventricle (LV). If the placement of the electrodes gave rise to AF, which was the case in all of the 1-week AT pigs, the AF was DC cardioverted (Zoll M Series, ZOLL Medical, Cheshire, United Kingdom) with the electrodes placed on the LA and RA using 5 to 30 J as appropriate. After conversion to SR, the heart was allowed 30 minutes of stabilization.

A programmable stimulator (Hugo Sachs, Germany) was used to deliver 2-ms double-rheobase current pulses, and the LA effective refractory period (ERP), the LV ERP, and the mean AF duration after burst pacing were measured. The LV ERP and LA ERP were measured at a basic cycle length of 400 ms. The ERP was determined as the longest S1-S2 failing to capture, with 10 basic stimuli (S1) followed by a premature extra stimulus (S2) with 5 ms increments. AF was induced by burst pacing at 50 Hz and 10 V. The mean AF duration was based on 10 AF inductions in each pig or 5 AF inductions if the mean AF duration of the first 5 AF durations was >1 minute and <5 minutes or as many AF inductions as time allowed (usually 1 or 2) within 30 minutes if the AF durations lasted >5 minutes. In 12 of 14 1-week AT pigs AF was induced by the first S2 stimulus after the LA ERP and was sustained for at least 30 minutes after which drug treatment was started.

Before the experiments, each pig was randomly assigned to receive either AP14145 or vernakalant 30 minutes after induction of the first AF episode.

Vernakalant was given as an infusion of 0.12 mg/kg per minute (60 minutes) followed by a maintenance infusion of 0.08 mg/kg per minute to produce a steady plasma concentration of ≈4000 ng/mL as described in ref ^21^, equivalent to the maximal plasma concentration measured in patients in the ACT I clinical trial (Arrhythmia Conversion Trial).^22^ Recordings after baseline in the groups receiving vernakalant were all conducted at steady state plasma concentration of vernakalant. AP14145 was given as 3 consecutive bolus injections for 1 to 2 minutes of 5, 8, and 8 mg/kg with 30 minutes intervals. Recordings in the groups receiving AP14145 were conducted a few minutes after each bolus dose.

### Cardioversion in Conscious Pigs

**Figure 1.**
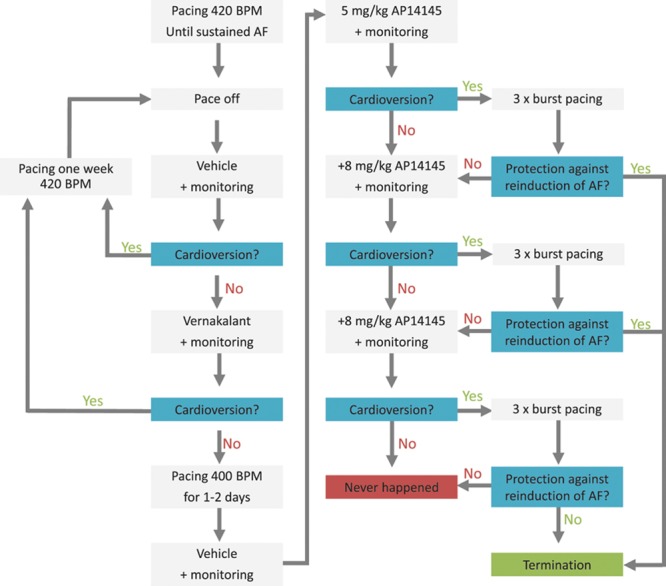
**Flowchart for cardioversion of vernakalant-resistant atrial fibrillation (AF) in conscious pigs.** BPM indicates beats per minute.

In the long-term AT pigs, after 7 days of recovery, the pacemakers were turned on and the RA was paced at 420 BPM (Figure 1). The pigs were medicated with digoxin (250 µg/d) starting 4 days before turning on the pacemaker. After 7 days pacing, the pigs were Holter-monitored (Televet 100; Engel Engineering Service GmbH, Heusenstamm, Germany) while the pacemaker was turned off. Sustained AF was defined as AF that was sustained after the pacemaker was turned off during >10 minutes monitoring and an additional 10 minutes with infusion of saline (1 mL/kg) followed by 30 minutes monitoring, a total of 50 minutes of AF. When sustained AF had been confirmed, vernakalant (4 mg/kg) was infused for 10 minutes, and the pig was monitored for an additional 30 minutes. If AF converted at any point, the pig was paced 420 BPM for another 7 days, and the procedure was repeated. When AF could no longer be converted to SR by vernakalant, the pig was paced 420 BPM for another 24 to 48 hours to allow vernakalant to be cleared from the system (half-life, 2–3 hours). After this, the pacemaker was turned off under Holter-monitoring. After having observed 10 minutes of AF, 1 mL/kg vehicle was injected over 2 minutes, and the ECG was monitored for 30 minutes. When AF did not convert to SR within this period, 5 mg/kg AP14145 was injected over 2 minutes, and the ECG was monitored for 30 minutes. If AF converted within 30 minutes, burst pacing with 50 Hz was given thrice. If AF did not convert or if a single AF episode lasting for >10 minutes could be induced with burst pacing, an additional 8 mg/kg AP14145 was injected over 2 minutes and the ECG was monitored for 30 minutes. If AF converted within 30 minutes, burst pacing with 50 Hz for 5 to 15 seconds was given thrice. If AF did not convert or an AF episode of at least 10 minutes duration could be induced with burst pacing, a final dose of 8 mg/kg AP14145 was injected over 2 minutes and the ECG was monitored for another 30 minutes. If at any point AP14145 injection gave rise to unexpected adverse effects, the pig was sedated by a small dose of zoletil while the experiment was completed. If at any point AF was converted and could not be reinduced with burst pacing, the experiment was terminated. Cardioversion was considered successful if AF was converted. Protection against reinduction of AF was considered successful if AF lasting >10 minutes could not be reinduced by burst pacing.

### Pharmacokinetic Measurements

Two pigs underwent pharmacokinetic studies and were pre-medicated, anaesthetized, intubated, and ventilated as described above. Paco_2_, temperature, and ECG were monitored. Invasive blood pressure was measured in the right femoral artery, and the pig was given 6 mL/kg per hour isotonic salt solution in an ear vein. At T=0, 5 mg/kg AP14145 was infused in the left external jugular vein, and blood samples were taken from the right femoral vein at (in minutes) T=−10, 5, 10, 15, 20, 30, 40, 60, 80, 100, 140, and 180 and stored in heparin treated tubes. Blood samples were centrifuged at 2G for 5 minutes at 4°C, and the plasma samples analyzed for AP14145 concentration at CiToxLAB ScanTox AS, Ejby, Denmark.

### *KCNNx* Expression in Pig Hearts

Cardiac tissue from each of the 4 heart chambers was obtained from long-term AT pigs (n=6) or healthy controls (HC; n=6). After pre-medication with zoletil pig mixture (250 mg dry tiletamin+zolazepam, 6.5 mL xylazine 20 mg/mL, 1.25 ketamine 100 mg/mL, 2.5 mL butorphanol 10 mg/mL, and 2 mL methadone 10 mg/mL) 0.1 mL/ kg given intramuscularly, the pigs were euthanized by intravenous injection of pentobarbital 200 mg/mL, and the hearts were excised and placed in ice-cold cardioplegic solution (NaCl 110.0, KCl 16.0, MgCl_2_ 16.0, CaCl_2_ 1.2, and NaHCO3 10.0 mmol/L). Total RNA from tissue samples was extracted according to manufacturer’s instruction (miRNeasy Mini Kit, Qiagen, CA). RNA was reverse transcribed to cDNA using the nanoscript2 kit (Primerdesign, United Kingdom). Expression profiling of the SK channels (*KCNN1*, *KCNN2*, and *KCNN3*) was performed using quantitative real-time quantitative polymerase chain reaction with Taqman double dye probes (Primerdesign, United Kingdom). For further details, please see Data Supplement.

### Data and Statistical Analyses

GraphPad Prism software (GraphPad Software, San Diego, CA) and Chart 7 software (ADinstruments) were used for data analyses and figures. Continuous data other than AF durations were summarized using the mean±SEM. AF durations were log-transformed to pass a Kolmogorov–Smirnov normality test. In the text, AF duration was given as a back transformation the mean of log AF duration (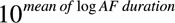) followed by the interval (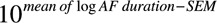
−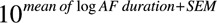) in parentheses. In figures, the AF duration is shown as the raw AF duration on a logarithmic scale. Expression data were log-transformed to obtain a more Gaussian distribution. In figures, the expression data are shown as the raw values on a logarithmic scale.

Wilcoxon matched pairs signed-rank test was used to compare pooled expression data from AF and HC pigs for LA versus LV and RA versus right ventricle. Mann–Whitney test was used to compare the differences in expression of KCNN2 and KCNN3 in AF versus HC, as well as LA ERP, LV ERP, and AF durations of CTRL and 1-week AT groups. Fisher exact test was used to compare the number of pigs with AF durations >30 minutes in the CTRL and 1-week AT groups. Ordinary 1-way ANOVA and Sidak correction for multiple comparisons test were used to compare mean values of heart rates in 1-week AT before AT, right after AT, and at vernakalant-resistant AF, as well as for comparing LA ERP, LV ERP, and log AF duration by concentration of AP14145 and vernakalant as compared with the respective baseline values.

*P*values are given with 3 significant digits, and values of *P*<0.05 were considered statistically significant.

### Compounds and Solution

Vernakalant (Brinavess) was purchased from a Danish pharmacy as a 20-mg/mL solution for intravenous infusion. Before infusion, it was diluted in sterile isotonic saline to yield a final concentration of 4 mg/mL. AP14145 is an Acesion Pharma proprietary compound and was synthesized at Syngene International, Bangalore, India. AP14145 was dissolved in dimethyl sulfoxide for cellular electrophysiology or saline and 30% hydroxypropylbetacyclodextrin (Roquette, France) for pig experiments. The solution was sterile filtered before use.

## Results

### AP14145: a New Small Molecule SK Channel Inhibitor

**Figure 2.**
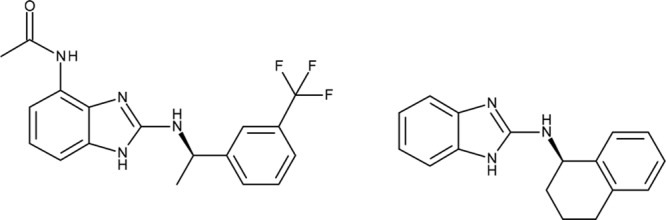
**Small-conductance Ca^2+^-activated K+** **channel inhibitors AP14145 (left) and NS8593 (right).**

The small molecule compound AP14145 (*N*-(2-{[(1*R*)-1-[3-(trifluoromethyl)phenyl]ethyl]amino}-1*H*-1,3-benzodiazol-4-yl) acetamide (Figure 2) was developed as a novel potent SK3 channel inhibitor and has structural similarities to the earlier published SK channel inhibitor NS8593.^23^

**Figure 3.**
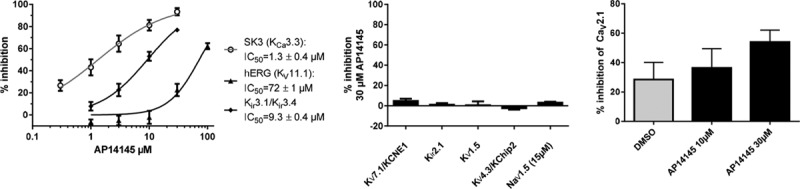
**Effect of AP14145 on heterologous expressed ion channels.** **Left**, Concentration response curves of AP14145 effects on hSK3 (n=11), hERG (n=5), and K_ir_3.1/K_ir_3.4 (n=6). **Middle**, % Inhibition of K_V_7.1/KCNE1 (n=9), K_ir_2.1 (n=5), K_V_1.5 (n=5), and K_V_4.3/KChiP2 (n=5) by 30 µmol/L AP14145 and on Na_V_1.5 by 15 µmol/L AP14145 (n=3). **Right**, % Inhibition of hCa_V_1.2 by dimethyl sulfoxide (n=3) or AP14145 by 10 and 30 µmol/L (n=6). hERG, NaV1.5, CaV1.2, and SK3 are measured by automated patch clamping and the remaining by 2-electrode voltage-clamp.

AP14145 demonstrated an IC_50_ in whole-cell patch clamp on the human SK3 channel of 1.3±0.4 µmol/L with a Hill-slope of 1.4±0.7 showing no cooperativity (Figure 3, left). To investigate the selectivity of AP14145, the effect of the compound was tested on a panel of cardiac ion channels (Figure I in the Data Supplement). AP14145 inhibited hERG (K_V_11.1) with an IC_50_ of 71.8±0.5 µmol/L and K_ir_3.1/K_ir_3.4 (*I*_KACh_) with an IC_50_ of 9.3±0.4 µmol/L (Figure 3, left) and did not produce any significant effects on K_V_1.5 (*I*_Kur_), K_V_7.1/KCNE1 (*I*_Ks_), K_V_4.3/KChiP2 (*I*_to_), and K_ir_2.1 (*I*_K1_) in 30 µmol/L or on Na_V_1.5 (15 µmol/L; *I*_Na_; Figure 3, middle). Concentrations from 1 to 10 µmol/L AP14145 produced no significant block of Ca_V_1.2, whereas AP14145 at 30 µmol/L changed the baseline significantly more than dimethyl sulfoxide (54±3% versus 28±7%; Figure 3, right).

### SK2 and SK3 Are Expressed in Pig Atria

The expression of SK channels in porcine heart has not been previously described. To further substantiate that effects of AP14145 on atrial electrophysiology were mediated by SK channels, we investigated the expression profile of *KCNN1*, *KCNN2*, and *KCNN3* (SK1–3) in the hearts of pigs with sustained AF, as well as controls. *KCNN1* was only found in diminutive amounts in all chambers of both HC and AF pigs (data not shown).

**Figure 4.**
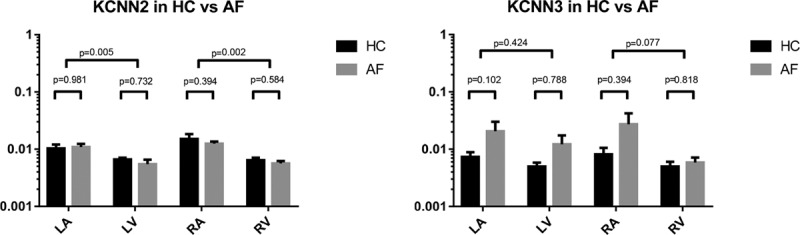
**Expression of KCNN2 (left) and KCNN3 (right) in tissue from the 4 heart chambers isolated from healthy control (HC) pigs (n=6) and pigs with sustained atrial fibrillation (AF;n=6).** LA indicates left atrial; and LV, left ventricle; RA, right atrial; and RV, right ventricle.

*KCNN2* was expressed in both atria and ventricles (Figure 4, left). The expression levels of *KCNN2* were not found to be significantly affected by tachypacing and the presence of AF in any of the chambers. A higher level (≈2-fold) of *KCNN2* was found in LA compared with LV (*P*=0.005) and in RA compared with right ventricle (*P*=0.002) in HC and AF pigs.

*KCNN3* was expressed in both atria and ventricles (Figure 4, right). The expression levels of *KCNN3* were not found to be significantly affected by tachypacing and the presence of AF in any of the chambers. No statistically significant difference between LA compared with LV (*P*=0.424) or RA compared with right ventricle (*P*=0.077) was observed.

### Pharmacokinetic Results

**Figure 5.**
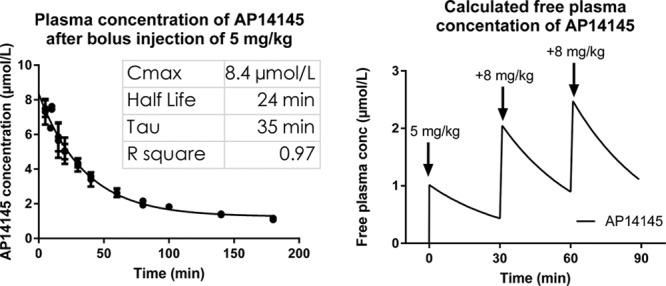
**Plasma concentrations of AP14145 in pig.** **Left**, Measured total plasma concentration of AP14145 in pigs after bolus injection of 5 mg/kg AP14145 fitted with an exponential 1-phase decay model. **Right**, Calculated free plasma concentration of AP14145 based on a plasma protein binding of 88% after 3 injections of 5, 8, and 8 mg/kg of AP14145 with 30-min intervals.

An exponential 1-phase decay model fitted the observations made with regard to plasma concentration of AP14145 over time with *R*^2^=0.97, C_max_=8355 nmol/L, t_½_=24.3 minutes, and τ=35 minutes (Figure 5, left). Using these values and the formula 
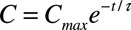
, as well as a plasma protein binding from minipig plasma of 88%, the free plasma concentrations of AP14145 at any given time during the experiments can be calculated for the dosing regimen used (Figure 5, right). According to these calculations, the maximal free plasma concentrations of AP14145 after each bolus injection were 1.0, 2.0, and 2.5 µmol/L (Figure 5, right), corresponding to 43%, 54%, and 67% *I*_KCa_-inhibition, assuming Michaelis–Menten kinetics.

### Open Chest Experiments: 1-Week AT and CTRL Pigs

#### Effects of 1-Week AT

Neither the LA ERP of 69±8 ms nor the LV ERP of 183±12 ms in the 1-week AT pigs was significantly different from their CTRL counterparts of 75±8 and 181±6 ms, respectively (*P*=0.517 for LA ERP and *P*=0.859 for LV ERP). However, the 1-week AT gave rise to significantly longer AF durations as compared with the CTRL with mean AF durations of 1130 seconds (822–1553 seconds) and 175 seconds (93–329 seconds), respectively (*P*=0.004). Significantly, more pigs in the 1-week AT group (10 of 12) had AF durations >30 minutes than in the CTRL group (2 of 9; *P*=0.009). Despite treatment with digoxin, 1-week AT increased the mean ventricular rate from 174±6 to 258±19 BPM right after initiation of AT (*P*<0.003), and the mean ventricular rate remained elevated (221±13 BPM; *P*=0.028) also after long-term AT when vernakalant-resistant AF was present.

#### Effects of Compounds in CTRL Pigs

**Figure 6.**
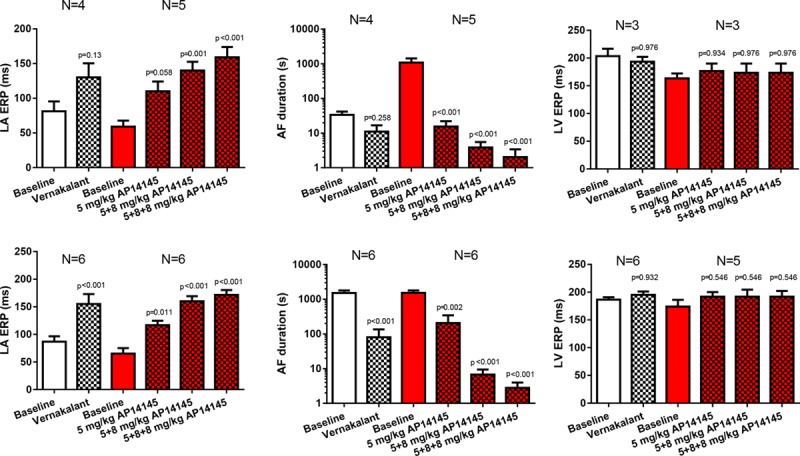
**Effects of vernakalant and AP14145 on left atrial (LA) effective refractory period (ERP), atrial fibrillation (AF) duration, and left ventricle (LV) ERP.** **Top**, Effects in control pigs. **Bottom**, Effects in 1-wk atrial tachypacing pigs.

In the CTRL pigs, both vernakalant and the low dose of AP14145 failed to significantly increase the LA ERP (Table 1; Figure 6, top). The higher doses of AP14145 caused a significant increase of LA ERP. The increase in LA ERP with AP14145 was dose dependent. Concomitant with the increase in LA ERP, the AF duration was decreased by AP14145. Again, the effect of AP14145 on AF duration was dose dependent. Neither vernakalant nor AP14145 changed the LV ERP significantly.

**Table 1.**
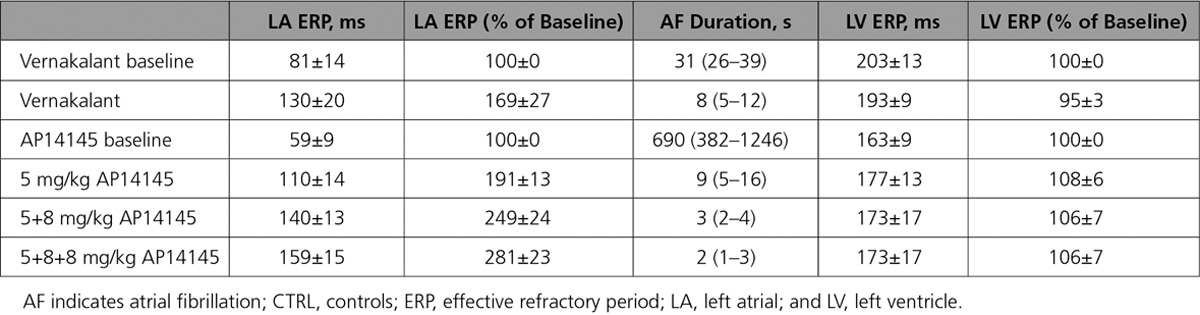
Effects of Vernakalant and AP14145 in CTRL Pigs

#### Effects of Compounds in 1-Week AT Pigs

The effects of vernakalant and AP14145 were comparable in both 1-week AT and CTRL groups; both compounds increased LA ERP and decreased AF durations with no significant changes of LV ERP (Table 2; Figure 6, bottom). The effects of AP14145 on LA ERP and AF duration were dose dependent.

In 5 of 6 pigs assigned to receive vernakalant and in 5 of 6 pigs assigned to receive AP14145, the AF that was induced during baseline recording lasted for >30 minutes, and the infusion of the compounds was initiated while AF was still ongoing. Both vernakalant and the lowest dose of AP14145 terminated the AF in 5 of 6 pigs in each 1-week AT group. The time to conversion was 13.8±5.5 minutes in the vernakalant group and 3.6±1.8 minutes in the AP14145 group.

### Cardioversion in Conscious Pigs

**Figure 7.**
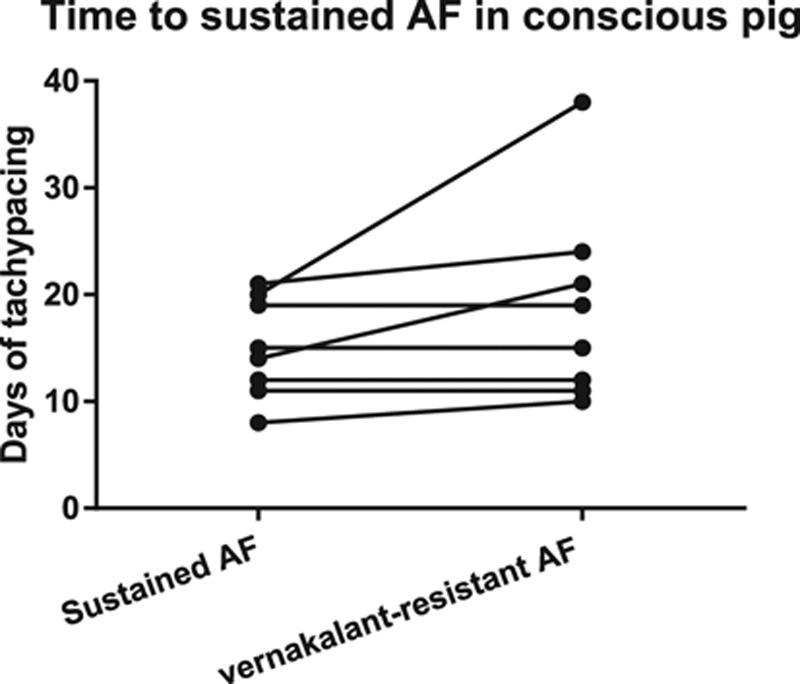
**Time to sustained atrial fibrillation (AF) in conscious pig.** Sustained AF was defined as AF that was sustained after the pacemaker was turned off during >10 min monitoring and an additional 10 min with infusion of saline (1 mL/kg) followed by 30 min monitoring, a total of 50 min of AF. Vernakalant-resistant AF was defined as sustained AF where vernakalant (4 mg/kg) infused for 10 min did not cardiovert AF to sinus rhythm during a monitoring phase of an additional 30 min.

**Figure 8.**
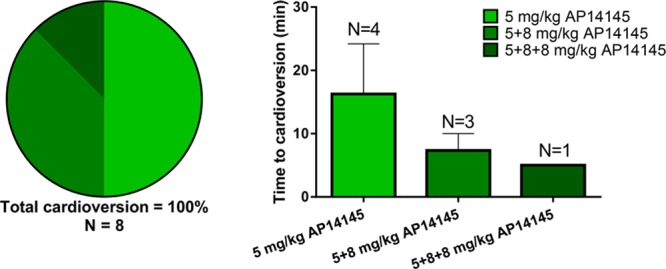
**Cardioversion and protection against reinduction of vernakalant-resistant atrial fibrillation (AF) with AP14145.** **Left**, Increasing doses of AP14145 cardioverts vernakalant-resistant AF and protects against reinduction of AF in 8 of 8 vernakalant-resistant AF pigs. **Right**, Time to cardioversion of AF by increasing doses of AP14145 in vernakalant-resistant AF pigs.

The average time for the development of sustained AF was 15.6±1.7 days of AT (Figure 7). On confirming sustained AF, the pigs received an infusion of 4 mg/kg vernakalant for 10 minutes and were observed for 30 minutes. In 2 of 8 pigs, AF was converted to SR, whereas AF remained unchanged in 6 of 8 pigs. The 2 pigs with conversion were tachypaced for an additional 1 and 2 weeks, respectively, before they became resistant to vernakalant. On average, vernakalant-resistant AF was obtained after pacing for 18.8±3.3 days. One to 2 days after having observed vernakalant-resistant AF, 8 of 8 pigs still had sustained AF when the pacemaker was turned off. Vernakalant-resistant, sustained AF was cardioverted to normal SR by 5 mg/kg of AP14145 in 4 of the 8 pigs, by an additional 8 mg/kg in 3 of the remaining 4 pigs, and with yet an additional dose of 8 mg/kg AP14145 in the last pig (Figure 8, left). Sustained AF (>10 minutes) could not be reinduced by burst pacing in any of the pigs 2 to 20 minutes after SR was restored. The time to conversion decreased with increasing doses of AP14145, from 16.3±7.9 minutes after 5 mg/kg AP14145 to 7.3±2.7 minutes after 8+5 mg/kg AP14145 to 5.0 minutes after 5+8+8 mg/kg AP14145 (Figure 8, right).

## Discussion

We present the first results from a model of vernakalant-resistant AF in conscious pigs showing that SK channel inhibition by AP14145 is capable of both converting vernakalant-resistant AF to SR and protect against reinduction of AF. Complementary molecular, biophysical, and molecular methods were used and showed that (1) SK2 and SK3 channels are present in the porcine atria in both HC and in pigs with vernakalant-resistant AF, (2) AP14145 is a reasonably potent and selective inhibitor of the SK3 current, and (3) SK channel inhibition by AP14145 selectively prolongs the refractory period in porcine LA but not in the LV.

SK channels are predominantly expressed in the atria compared with the ventricles in the normal heart of several species, including humans, with no differences between LA and RA.^11,24–26^ Functionally, SK channels play a greater role in the atria compared with the ventricles when using effect criteria, such as ERP prolongation, action potential durations, and QT intervals.^8,11,16^ These findings have led to the notion that SK channels might be an interesting target for development of novel treatment for AF where more atrial selective pharmacotherapy has long been sought for. Significant increases of both LA ERP and RA ERP without similar effects in the LV ERP or ventricular repolarization are promising results relating to both the efficacy and safety of the treatment.

### Expression of Porcine Cardiac SK Channels

We present, for the first time, evidence that SK2 and SK3 but not SK1 subunits are expressed in porcine atria and ventricles in HC pigs and in pigs with vernakalant-resistant AF. Skibsbye et al^16^ found that the SK1 subunit was minimally expressed in RA appendage tissue from patients in SR, as well as patients with >6 months in AF, but both the SK2 and SK3 channel subunits were present in SR tissue as well as chronic AF tissue, although to a significantly lower extent in the latter. Considering the SK channel subtypes present in the atria, the pig seems to be more similar to man than, for example, the dog, where all 3 SK channel subtypes are present in the atria.^11^ According to the quantitative polymerase chain reaction data from the current study, SK channel mRNA was expressed in slightly higher amounts in both atria compared with the ventricles of both the control pigs and pigs with long-term AT. Although a tendency toward an increased SK3 expression in pigs with long-term AT compared with HC pigs was observed in tissue from LA, RA, and to a lesser extent LV, this apparent upregulation of SK3 was not statistically significant.

### Effects of AP14145 in Pigs

AP14145 is a new SK channel inhibitor that demonstrated a reasonable selectivity toward cardiac SK channels compared with the other cardiac ion channels tested. The functional data from our open chest experiments suggest an atrial selective effect of AP14145. The LA ERP was concentration-dependently increased almost 3-fold by AP14145 while the LV ERP remained unchanged in both control pigs and pigs that underwent 1 week of AT. One explanation for this atrial selectivity of AP14145 could be differences in expression levels. As described above, SK channel mRNA was expressed in slightly higher amounts in the LA compared with the LV of both CTRL pigs and pigs with vernakalant-resistant AF. Other potentially contributing explanations for this apparent atrial selective effect include Ca^2+^ dynamics in ventricles versus atria, location of the channels and neighboring L-type Ca^2+^ channels, transportation to the membrane, and redundancy of other repolarizing currents in the ventricles that renders SK current relatively less important in the ventricles. These possibilities were not investigated in the present study.

The calculated peak free plasma concentration of AP14145 was ≈1, ≈2, and ≈2.5 µmol/L after each injection, whereas the infusion regimen of vernakalant that was used should produce a steady state free unbound concentration of 4.8 µmol/L based on a total plasma concentration of 4000 ng/mL,^21^ a molar weight of 349.5 g/mol, and assuming a plasma protein binding of 58% based on the human plasma protein binding of 53% to 63%.^27^ As expected, vernakalant increased the LA ERP and reduced AF duration in the open chest experiments in both CTRL and 1-week AT pigs. In these settings of no or little remodeling, the efficacy of vernakalant (4.8 µmol/L free) and the lowest dose of AP14145 (1 µmol/L free) on AF duration and LA ERP were comparable. The highest dose of AP14145 had more prominent effects on LA ERP and AF duration even though the maximal free plasma concentration of AP14145 was only half of that of vernakalant.

In the long-term AT conscious pigs, vernakalant was administered as an infusion of 4 mg/kg given over 10 minutes. In the clinical phase I study of vernakalant, this dose resulted in a C_max_ of 2.0 to 2.2 µg/mL which corresponds to a free unbound C_max_ of ≈3.5 µmol/L which is slightly more than the maximal plasma concentration of AP14145 that was used. The results of vernakalant and AP14145 in the long-term AT pigs were different. AP14145 converted the vernakalant-resistant AF to SR in all 8 pigs tested. We cannot make firm conclusions on the mechanism behind the vernakalant-resistance, but it is likely not because of reduced expression of its main ion channel targets as we found that the expressions of K_V_1.5 and Na_V_1.5 were not affected by AT (data not shown). This in concordance with other studies suggesting that the loss of cardioversion efficacy of vernakalant is likely not due at decreased effect of vernakalant on its target ionic currents but rather because of other AF stabilizing processes.^28,29^

### AT and Cardiac Remodeling

The atrial selective effect observed in this study might be changed by certain pathophysiological conditions, such as myocardial infarction and congestive heart failure. Under these conditions, some studies have shown that ventricular SK current can be upregulated and that blocking SK current can exhibit both proarrhythmic^30,31^ and antiarrhythmic^32,33^ ventricular effects, depending on the experimental setup.

The main focus of this study has been to examine whether SK channel inhibition could be beneficial in a setting of relatively early persistent AF with remodeling to a degree that renders pharmacotherapy for recent onset AF ineffective. Even though all the pigs undergoing AT received digoxin to prevent symptoms of heart failure, there might be a component of ventricular remodeling in our pig model of AF because the ventricular rate was increased during tachypacing. However, no symptoms of heart failure were observed. Also no signs of ventricular proarrhythmia in the presence of AP14145 were observed in any of the tested pigs. Whether SK channel inhibition can have any ventricular effects in pigs under pathophysiological conditions, such as myocardial infarction and congestive heart failure, thus remains an open question.

In patients with permanent (>6 months) AF, the atrial effective refractory period (AERP) is shortened, but in RA tissue from patients with paroxysmal AF, this does not seem to be the case.^34–37^ A recent study has revealed a correlation of the degree of left ventricular dysfunction to prolongation of atrial refractoriness, showing that decreased left ventricular function is associated with relative action potential duration prolongation in patients with SR, paroxysmal AF, as well as permanent AF.^37^

In our short-term AT group, 1 week of pacing increased the susceptibility to AF but changed the refractory periods in neither atria nor ventricles compared with CTRL.

The absent change in AERP after short-term AT is different from what has been observed in dogs, goats, and sheep, where short-term tachypacing has been shown to significantly decrease atrial refractoriness.^3,11,38^ This discrepancy could be caused by species differences or ventricular remodeling resulting in decreased left ventricular function. In the animal models demonstrating abbreviated AERP, no signs of decreased left ventricular function were reported. In the dog and sheep models of AF, the atrioventricular node had been ablated, and in goats, AF does not seem to cause ventricular dysfunction.^39^ In a dog model of AF, in the presence of congestive heart failure caused by ventricular tachypacing, the decrease in AERP was smaller (24%) than in a model of AF with no congestive heart failure (42%).^40^ Likewise, in a pig model of AF with congestive heart failure induced by AT, no reduction of AERP was reported after 7 days of pacing—on the contrary, a significant AERP prolongation was observed.^41^ We cannot exclude a component of ventricular dysfunction in our model. Even though the pigs received digoxin to prevent symptoms of heart failure, the ventricular rate was significantly increased by AT, and the unchanged AERP value could therefore be speculated to result from the combination of tachypaced induced shorting of AERP and concomitant prolongation associated with ventricular dysfunction.

### AP14145 and Adverse Events

In the present study, we did not see any significant effects of AP14145 on the heart rate, the blood pressure, the ventricular repolarization (LV ERP), or the QRS interval. In all the conscious long-term AT pigs, adverse effects, such as vomiting, were seen 20 to 30 minutes after injection of AP14145. A dose of zoletil (a mixture of tiletamine hydrochloride and zolazepam hydrochloride) was used to remedy these effects but in effect left the pigs unconscious. Two of the 8 vernakalant-resistant pigs cardioverted on AP14145 before zoletil was injected, but in 6 of 8 pigs zoletil had been injected 2 to 40 minutes before cardioversion was obtained. We cannot exclude that AP14145 had been better tolerated if it had been given as an infusion over longer time.

### Limitations

After the higher doses of AP14145, cardioversion was obtained within 10 minutes in all pigs, but we cannot rule out a possible cardiac impact of zoletil in 6 of the 8 pigs. We cannot exclude that other effects of AP14145 than SK channel inhibition could have influenced our findings. However, AP14145 did not affect a panel of relevant cardiac ion channels (Figure 3) and had a 65- and 8-fold higher IC_50_ on hERG and IK_ACh_ as compared with SK3. Moreover, based on the measured free plasma concentrations of AP14145 in our experiments, we do not anticipate a significant contribution from inhibition of hERG or IK_ACh_. The small sample sizes for the quantitative polymerase chain reaction experiments and for the open chest CTRL pigs increase the chance of a type II error.

### Summary and Conclusions

In healthy pigs and pigs that had been subjected to 1-week AT, both vernakalant and AP14145 prolonged the AERP and reduced the duration of acutely induced AF while not significantly prolonging the LV ERP or showing other signs of ventricular effects. In an advanced pig model of long-term AT-induced sustained AF where clinically relevant doses of vernakalant could no longer convert the AF to SR, AP14145 was able to convert the pigs to SR and protect against reinduction of AF. Vomiting needing attention occurred after all doses of AP14145 that produced conversion to SR in pigs. The capability of SK channel inhibition to prolong atrial but not ventricular refractoriness and to convert vernakalant-resistant AF, as well as protecting from reinduction of AF, was confirmed in pigs with the tool compound AP14145. SK channel inhibition is an interesting concept and a promising target for development of new treatment of AF.

**Table 2.**
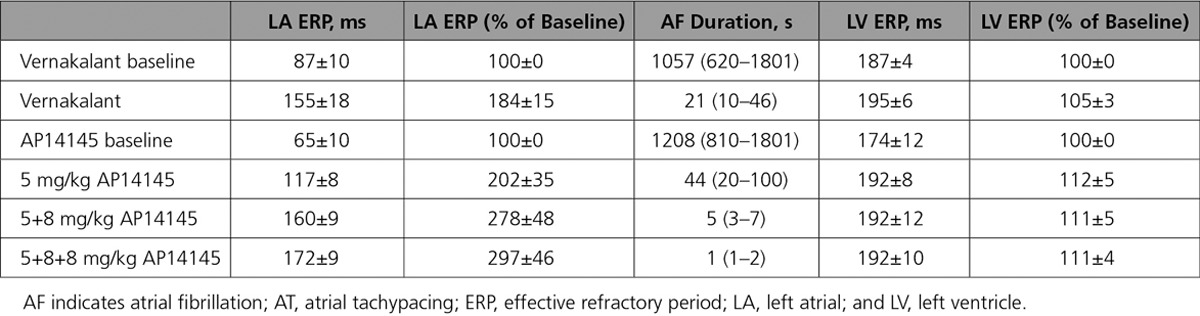
Effects of Vernakalant and AP14145 in 1-Week AT Pigs

## Acknowledgments

We thank Amer Mujezinovic and Lea Abildgaard for expert technical assistance.

## Sources of Funding

The study was supported by Innovation Fund Denmark, the Carlsberg Foundation, the Wellcome Trust (award reference no. 100406/Z/12/Z), and the European Union’s Horizon 2020 research and innovation programme under the Marie Skłodowska-Curie grant agreement no. 675351.

## Disclosures

Drs Diness, Sørensen, Grunnet, and Bentzen are used by and have interests in Acesion Pharma and are inventors of Acesion Pharma patents within the field of SK channels. The other authors report no conflicts.

## Supplementary Material
