# Supplementary material for: Termination of Vernakalant-Resistant Atrial Fibrillation by Inhibition of Small-Conductance Ca2+-Activated K+ Channels in Pigs
Source: Circ Arrhythm Electrophysiol. 2017 Oct 17;10(10):e005125. doi: 10.1161/CIRCEP.117.005125 (PMC5647113; doi:10.1161/CIRCEP.117.005125)
Supplement: Supplementary file 1 [file hae-10-e005125-s001.pdf]

Supplemental material

In vitro electrophysiology

Automated patch clamp experiments

SK, hERG, NaV1.5 and CaV1.2 experiments were performed using a QPatch 16 HT system and single-hole Qplates (Biolin Scientific, Sophion, Denmark). The Qpatch generates giga sealing, whole-cell formation, compound application and recording of current automatically.

SK assay

Automated whole-cell patch-clamping was performed on HEK-293 cells stably expressing $hK_{Ca}2.3$, in symmetrical K^+ solutions, with an extracellular solution consisting of (in mM): KCl 150; $CaCl_2$ 0.1; $MgCl_2$ 3; HEPES 10; Glucose 10, pH=7.4 with KOH, and an intracellular solution consisting of (in mM): KCl 108; KOH/EGTA 31.25/10; $CaCl_2$ 8.1; $MgCl_2$ 1.2; HEPES 10; KOH 15, pH adjusted to pH=7.2 with HCl. The free calcium concentration was calculated to 400 nM. The cells were clamped at 0 mV and $hK_{Ca}2.3$ currents were continuously elicited every 5th second by a linear voltage ramp from –80 mV to +80 mV (200 ms in duration). The compound application protocol consisted of 9 recording periods lasting from 50-200 s: 1) Baseline recordings in extracellular solution; 2) Application of the positive control methyl-bicuculline (100µM) (Sigma), which is characterized by full efficacy, fast on- and off-rate; 3-4) Wash-out; 5-9) Increasing concentrations of test compound to establish an IC_{50} value. Data were sampled at 10 kHz, 4th order Bessel filter, cut-off frequency 3 kHz. Currents were compensated for run-down. Potency was quantified as the concentration needed to inhibit half of the SK channel activity recorded at -80 mV and reported as an IC_{50} value. All drug effects were normalized to the observed full inhibitory effect of methyl-bicuculine.

hERG Assay

Recordings were performed on CHO-K1 cells stably expressing hKV11.1 with an intracellular solution containing (mM): CaCl2 5.4; MgCl 1.75; KOH/EGTA 31.25/10; KCl 120; HEPES 10; Na2ATP 4, pH pH=7.2 with KOH, and an extracellular solution (mM): CaCl2 2; MgCl2 1; NaCl 145; KCl 4, HEPES 10; Glucose 10, pH=7.4 with KOH. The cells were kept in voltage clamp and held at -90 mV. Currents were elicited every 7th ‘s second by depolarizing the membrane potential to +20 mV for 2 s followed by a 2 s voltage step to -50 mV in order to record tail currents. The application protocol used was: baseline recordings in extracellular solution followed by application of increasing concentration of AP14145 (1, 3, 10, 30, 100 µM). Finally 100µM Dofetilide was added as a positive control. Effect of AP14145 on tail current amplitude was

recorded at baseline and following drug application, normalized to full block by dofetilide and used to establish the IC_{50}. Data were sampled at 10 kHz, 8th order Bessel filter, cut-off frequency 3 kHz, and 80% Rs compensation. Only experiments with a whole cell seal of > 500 MΩ were used.

NaV1.5 assay

The effect of AP14145 was examined using HEK293 cells stably expressing rNaV1.5. NaV1.5 currents were recorded with an intracellular solution that contained (in mM) 135 CsF, 1/5 EGTA/CsOH, 10 HEPES, 10 NaCl, 4 Na-ATP, pH 7.3, 300 mOsm and an extracellular solution containing (in mM): 2 $CaCl_2$, 1 $MgCl_2$, 10 HEPES, 4 KCl, 145 NaCl, 10 glucose, pH 7.2, 310 mOsm (adjusted with sucrose). rNaV1.5 currents were elicited every 1000 milliseconds by a 50 ms depolarizing step to -20 mV from a holding potential of -120 mV (a total of 80 pulses). The application protocol used was baseline recordings in standard extracellular solution followed by application of AP14145 (15µM). The current amplitude at the 80th pulse was used for analysis. Data were sampled at 25 kHz, eighth-order Bessel filter, cutoff frequency 3 kHz. Only experiments with a whole-cell seal of >500 M[ohm] were used.

Calcium assay

Effect of AP14145 on L-type calcium channels was investigated by automated patch clamping (Qpatch, Sophion) using a hCaV 1.2 stable cell line (Alpha1C, Beta2C and Alpha2delta) (SB Drug Discovery).The extracellular solution contained (in mM): 145 NaCl, 10 BaCl2, 4 KCl, 10 HEPES and 10 Glucose (pH 7.4). The intracellular solution contained (in mM) 27 CsF, 112 CsCl, 2 NaCl, 8.2 EGTA, 10 HEPES and 4 MgATP; (pH 7.25). The stimulus regime consisted of an initial 50 ms pulse at -120 mV, followed by a 300 ms test pulse at 0mV, and then returned back to -120 mV for another 50 ms. Four cumulative concentrations of compound was applied to the cell (1, 3, 10, 30 (in µM)), followed by the positive control, Nifedipine (10µM).

Two-electrode voltage-clamp

cRNA for injection was prepared according to from linearized plasmids using the mMESSAGE mMACHINE T7 kit (Ambion, TX, USA). RNA concentrations and quality were assessed by UV spectroscopy (NanoDrop, Thermo Scientific,

Wilmington, USA) and gel electrophoresis. The following concentrations of cRNA were used (µg/µL): K_V7.1+KCNE1 0.08 + 0.02; K_{ir}2.1 0.4; K_V1.5 0,1; K_V4.3+ KChIP2 0.01+0.01; K_{ir}3.1+K_{ir}3.4 0.04+0.04. 50 nL cRNA was injected in *Xenopus laevis* oocytes (EcoCyte Bioscience, Castrop-Rauxel, Germany), and currents were recorded after 2-3 days of incubation (19°C) using a two-electrode voltage-clamp amplifier (Dagan CA-1B; IL, USA). Borosilicate glass recording electrodes (0.5–1 MΩ when filled with 2 M KCl) (Module Ohm, Herlev, Denmark) were pulled on a DMZ-Universal Puller (Zeitz Instruments, Martinsried, Germany). Oocytes were superfused with Kulori solution (in mM: NaCl 90, KCl 4, $MgCl_2$ 1, $CaCl_2$ 1, 4-(2-hydroxyethyl)-1-piperazineethanesulfonic acid 5, pH=7.4 with NaOH, room temperature). After stabilization the solution was changed for a solution containing AP14145 (30µM). For Kir3.1/kir3.4 four concentrations of AP14145 were tested (1, 3, 10, 30 µM) in order to establish an IC50 value.

Oocytes were voltage clamped at -80 mV. KV7.1/KCNE1 currents were elicited by depolarizing the membrane potential to +20 mV for 5 s followed by a 1 s step to -30 mV. KV1.5 and KV4.3/KChIP2 currents were evoked by changing the membrane potential to +20 mV or 0 mV for 2 s respectively. Steady state current amplitudes (KV7.1/KCNE1 and KV1.5) and peak current amplitudes (KV4.3/KChIP2) were used for analysis. Kir3.1/Kir3.4 and Kir2.1 currents were elicited by a ramp protocol from -100 mV to +80 mV (5 s duration). Current amplitudes at -100 mV were used for analysis.

Data acquisition was performed with the Pulse software (HEKA Elektronik, Lambrecht/Pfalz, Germany).

KCNNx expression in pig hearts

Tissue samples

After premedication with zoletil pig mixture (250 mg dry tiletamin+zolazepam, 6.5 ml xylazine 20 mg/ml, 1.25 ketamine 100 mg/ml, 2.5 ml butorphanol 10 mg/ml, and 2 ml methadone 10 mg/ml) 1 ml/10 kg given IM, the pigs were euthanized by intravenous injection of pentobarbital 200 mg/ml. The hearts were immediately excised and placed in ice-cold cardioplegic solution (NaCl 110.0 mM, KCl 16.0 mM, $MgCl_2$ 16.0 mM, $CaCl_2$ 1.2 mM, NaHCO3 10.0 mM). Cardiac tissue from each of the four chambers was obtained from long-term AT pigs (n=6) or controls (n=6) and rapidly snap-frozen in liquid nitrogen. Tissue was kept at -80°C for later RNA extraction.

Approximately 40mg of atrial and ventricular tissue specimens were lysed in QIAzol reagent (QIAGEN, Maryland, USA) using a Precellys 24 (Bertin Technologies, Montigny-le-Bretonneux, France). Total RNA including small RNAs was purified according to the manufacturer's instruction using the miRNeasy Mini kit (QIAGEN, Hilden, Germany). RNA samples were treated with DNAse. The RNA concentration and purity was determined by spectrophotometry (NanoDrop2000, Thermo Scientific, Wilmington, USA) using the absorbance ratio of A260/A280.

Expression profiling

Reverse transcription (RT) reactions were performed following the manufacturer's instructions using the Precision nanoScript2 Reverse Transcription kit (PrimerDesign Ltd., Southampton, UK). Each reaction had a final volume of 20 µL containing 1µg of total RNA. The experiment included a minus reverse transcriptase control (-RT) to check for genomic contamination and other amplification artifacts. The reverse transcription steps were conducted in a PTC-200 Peltier Thermal Cycler (Struers KEBO Lab, Albertslund, DK) as follows: 25°C for 5 minutes, 42°C for 20 minutes and 75°C for 10 minutes. To assess the levels of mRNAs from the genes KCNN1, KCNN2 and KCNN3 in the samples, quantitative real-time polymerase chain reaction (qPCR) was carried out in duplicates using Custom real-time PCR assays with double dye probe (Taqman style) and PrecisionPLUS MasterMix with ROX (PrimerDesign Ltd., Southampton, UK) according to manufacturer's instruction. *GAPDH* and *GPI* were used as reference genes for qPCR normalization and no template controls (NTCs) were run simultaneously to assess contamination. The qPCR steps were conducted with Precision BrightWhite real-time PCR 96-well plates (PrimerDesign Ltd., Southampton, UK) on a CFX Connect Real-Time System (BIO-RAD, Hertfordshire, UK) as follows: 95°C for 2min followed by 40 cycles of 95°C for 15 seconds and 60°C for 1 minute with FAM dye fluorescence read at the end of each cycle. Threshold cycle (C*t*) values were obtained using Bio-Rad CFX96 Managed 3.0 software and assuming a single threshold mode. The data was transferred to a spreadsheet for calculation of ΔC*t*s and the relative expression of the genes in both groups was calculated using the $2^{-\Delta Ct}$ method.

Primer sequences

Gene	Forward primer	Reverse primer	Product length (bp)	Accession number
KCNN1	5'- CTCCTGGATCATTGCTGCCT -3'	5'- TTGCTGGTCACTTCCTGCTT -3'	81	XM_005654898
KCNN2	5'- CGCCGCTTCGTCCTCAG -3'	5'- CCGCCTCCTCCAGTGCT -3'	111	XM_005661582
KCNN3	5'- ATCAGTCTGTCCACCATCATCC -3'	5'- CTCGTAGGTCATGGCTATCCG -3'	117	NM_213985

Reference Genes	Species	Accession number	Anchor Nucleotide	Product length (bp)
GAPDH	Sus scrofa	AF017079	918	188
GPI	Sus scrofa	NM_214330	685	105
